# Antisepsis in Vienna

**Published:** 1885-04-20

**Authors:** F. W. Rohr

**Affiliations:** Professor Billroth’s Clinic


### ANTISEPSIS IN VIENNA.

By F. W. Rohr, M.D., at Professor Billroth's Clinic.

After careful and manifold observations with the different antiseptics, iodoform has been given first place, and has, since 1881, formed the basis of the antiseptic treatment at this clinic. It displays its wonderful power in all cases in which the Lister method cannot be carried out, and seems to exercise a specific influence upon fungoid, lupoid and syphilitic processes.

1. Dressings for wounds having a tendency to heal per primam or by first intention.

In superficial wounds, such as on the face, no dressing is applied; however, in some cases, for safety's sake, such wounds are painted over with iodoform collodion, and in some instances a light iodoform dressing is applied. In deeper wounds the iodoform takes the place of the Lister dressing. After an operation is completed, the wound properly drained and closed and the surrounding tissue cleansed with a one-per-cent. carbolic solution, the wound is covered with from two to four layers of iodoform gauze, over which is placed, according to amount of secretion expected, a thicker or thinner layer of carbolic gauze, several layers of absorbent cotton, and lastly the whole is covered with a layer of Billroth-battist (a water-tight cloth), the same having been dipped in a one-per-cent. carbolic solution. Now a common bandage is applied, and in some cases in which compression is required several turns of starch bandage are applied.

2. Dressings for wounds not having a tendency to heal per primam.

For this class of cases iodoform is pre-eminently fitted, as with no other antiseptic such results can be obtained, large wounds healing without reaction and with very few changes of dressings. Such wounds, and especially those with cavities, are not united, but thoroughly irrigated and filled with iodoform gauze. In irregular cavities small strips of the gauze are introduced into every part over which others are loosely entered until the cavity is filled, when a few layers are so placed as to cover the wound and immediate surrounding tissue. The common bandage is now applied. Healing takes place by granulation, and instead of changing the dressing every day as by the old methods of treatment, they are allowed to remain from eight to fourteen days. In infected wounds, gangrenous ulcers, etc., iodoform produces a rapid cessation of the infection and its results, and produces healthy granulations. In cases of carcinoma it destroys the fetor better than any other remedy. In specific ulcerations of lupoid or tubercular origin it seems to act only locally on the infiltrations and granulations, and in these cases the powder should be dusted over the parts affected before applying the gauze.

A few words in regard to its application in fungoid processes and in wounds communicating with cavities covered by mucous membrane.

In fungoid processes in the soft tissues and bones (caries), it is not necessary after removal of the infected tissues and resection of the bone to have the whole wound open, but it may be almost wholly united by sutures, leaving only space for the secretions to drain away, and through which the iodoform gauze, which has been introduced, can be removed. In these cases, now, only the iodoform gauze is used, but in some cases, in which it is thought that more iodoform is indicated, the powder is first dusted into the wound and then the gauze introduced. It is also necessary in these cases that every part of the wound be covered with the gauze.

Small cold abscesses, which originate from the soft tissues or bones, which are accessible, heal nicely after being opened and dressed with iodoform. Abscesses arising from caries of the bones, which are not accessible, such as from the vertebræ or pelvic bones, are either left to open spontaneously or are punctured with a trocar and canula and injected with iodoform in suspension, generally glycerine. No cleansing with carbolized solution is necessary, but as soon as pus ceases to flow through the canula the iodoform in suspension is injected, so that the cavity of the abscess is about half filled. I have often seen as much as 6 grammes or 90 grains of iodoform injected into such abscesses with gratifying results. After the injection the puncture is closed with adhesive plaster, and a bandage applied. In some cases it is necessary to repeat the operation several times before a cure is effected. In wounds implicating the mucous membranes, such as the cavity of the mouth, œsophagus, rectum, vagina, urethra, etc., the so-called "Klibendi iodoform gauze" or adhesive gauze is used (iodoform et collophonium). It is used because the iodoform is not easily washed off by the mucous secretions, and also because, by virtue of its adhesiveness, there is no danger of its being swallowed if introduced into the cavity of the mouth. It also has another advantage, that of its being hemostatic, and serves a good purpose to check parenchymatous bleedings. The dressings are left intact in all cases, as before stated, for a period of from eight to fourteen days, unless symptoms of fever supervene. In cases of fever the wounds are examined to determine whether there is retention of the secretions, too much compression, too high tension of the sutures, etc., and in case any one or the other of these conditions is found to be present it is at once remedied. If, after this procedure, the symptoms of fever do not disappear, the secretions become putrid, which rarely occurs, the dressings are removed daily, and to avoid intoxication only very little iodoform gauze is used. The wound is first irrigated with a five per cent. carbolic solution. If, after this, fever still supervenes, which is rare, the disinfection of the wound is regarded as a failure, the sutures are removed and the wound filled with the gauze, or with simple gauze impregnated with Burow's solution.

The iodoform dressings are used only two or three weeks, or until granulation is progressing nicely, and the wound is dressed with a salve, principally zinc or boracic acid. In cases of flabby granulation a nitrate of silver ointment is used, or the granulations are touched with the caustic.

Iodoform is used as 1, powder; 2, gauze; 3, iodoform collodion (1:10); 4, iodoform glycerine (10 to 20:100); 5, iodoform bacilli, after the formula,

℞ Iodoform pulv. . . . . . . . . . . . . . . . . . . . . . . . . . . . . . 20.00
Gummi arabici }
Glycerini, } aa. . . . . . . . . . . . . . . . . . . . . . . . . . 2.00
Amyli, }
M. Fiant bacilli diversi magnitud.

6, iodoform vaseline (20 to 50:100).

Carbolic acid is used in one-per-cent., two-and-a-half per-cent. and five-per-cent. solutions. The one-per-cent. is used for washing the hands, irrigating fresh wounds, etc. The two-and-a-half-per-cent. is used for disinfecting instruments and suture materials. The five-per-cent. is used for cleansing wounds in which septic material has become lodged, and where the weaker solutions are thought inadmissible.

Besides these there is in use a ten-per-cent. carbolic glycerine, in which are immersed metal catheters, uterine sounds, etc. Just before using them they are washed off with a one-per-cent. solution to prevent irritation.

Another very good solution in use here is the so-called Burow's solution:

℞ Alum crudi. . . . . . . . . . . . . . . . . . . . . . . . . . . . . . 5.00
Plumbi acet. . . . . . . . . . . . . . . . . . . . . . . . . . . . . 25.00
Aqua dest. . . . . . . . . . . . . . . . . . . . . . . . . . . . . 500.00 M.

I have seen excellent results from this solution, especially in phlegmonous inflammations, and if applied early in these cases it will prevent suppuration. Permanganate of potassium is also used, but only as a disinfectant for the hands, and for preparing sponges.

The most scrupulous cleanliness in regard to instruments and the hands of the operator and assistants is insisted upon. The hands are first thoroughly washed with soap and water, then dipped into a solution of potass. permang. (1:100), washed off with a ten-per-cent. solution of oxalic acid and finally dipped into a one-per-cent. carbolic solution.

In the obstetrical and gynecological wards, bichloride of mercury solution (1:1000) is used instead of the carbolic solution, and striking results are obtained from its use. In puerpural metritis, irrigation of the uterus with the bichloride solution is rigidly enforced and the mortality has been reduced to the minimum. In operations about the genitals irrigations with the bichloride solutions are made, and after the operations iodoform dressings are applied.

In the obstetrical wards every practitioner, before making a vaginal examination, must thoroughly wash his hands with soap and carbolized water, then disinfect again with solution permang. potass. This must be repeated before every examination.

It is owing to the rigid enforcement of these antiseptic precautions that there is so little septic infection among the six thousand women that are annually delivered in these wards.—*Chicago Med. Times.*

